# Vision–language guided semantic-geometric transformer for memory-efficient 3D scene understanding

**DOI:** 10.3389/frai.2026.1945529

**Published:** 2026-09-25

**Authors:** Licheng Liu, Yu Li, Fuyong Liu

**Affiliations:** 1 School of Electronic and Electrical Engineering, Wuhan Textile University, Wuhan, China; 2 School of Information Science and Engineering, Xinjiang College of Science and Technology, Korla, Xinjiang, China

**Keywords:** 3D scene understanding, memory-efficient transformer, point-cloud segmentation, semantic-geometric attention, vision–language models

## Abstract

Recent 3D Transformers have become a dominant framework for point-cloud segmentation by modeling spatial context in sparse 3D scenes. However, geometry and color alone provide limited high-level semantic cues, especially for cluttered boundary regions, visually similar objects, and long-tail categories. To address this issue, we propose a segmentation framework guided by Contrastive Language–Image Pre-training (CLIP) that enriches sparse 3D tokens with vision–language semantic priors. Specifically, dense CLIP features extracted from multi-view RGB images are projected onto 3D points through visibility-aware alignment and view pooling, and are fused with relative geometric offsets and color cues to form semantically aware sparse voxel tokens. To better exploit the aligned CLIP semantics during local token interactions, we build on contextual relative signal encoding (cRSE) and introduce a decoupled CLIP-induced semantic residual that forms semantic-geometric attention biases for local window attention. We further adapt block-wise online softmax computation to generate and consume these biases on the fly. Experiments on ScanNet, ScanNet200, and S3DIS demonstrate competitive segmentation performance, improved instance-level discrimination, and a 25.7% reduction in peak *online 3D-stage* training memory compared with the materialized attention implementation when cached CLIP features are used.

## 1 Introduction

3D scene understanding is fundamental to embodied intelligence, and point-cloud segmentation provides essential spatial-semantic representations for tasks such as scene mapping, robot navigation, and object interaction. To model complex spatial context in sparse 3D data, recent research has moved from point-based local aggregation methods (Zhao et al., 2021; Zhang et al., 2022) toward Transformer-based architectures (Lu et al., 2025; Wu et al., 2024; Lai et al., 2022; He et al., 2024; Han et al., 2024). In particular, window-based 3D Transformers (Yang et al., 2025a,b; Liu et al., 2021) have emerged as a practical design choice because localized attention achieves a favorable balance between efficiency and receptive field while preserving geometric inductive bias (Liu et al., 2023; Guo et al., 2022). However, their geometry-centric design still leaves high-level semantic understanding insufficient, especially in cluttered scenes (Akwensi et al., 2024; Kim et al., 2026; Li et al., 2024; Khan et al., 2024).

This semantic limitation arises from two factors. First, semantic cues in point clouds are sparse and ambiguous: geometry and optional color alone provide limited high-level semantic cues, making it difficult to maintain consistent predictions for visually similar structures, cluttered boundary regions, and long-tail categories across distant regions (Peng et al., 2023; Takmaz et al., 2023; Jatavallabhula et al., 2023; Tai et al., 2026). Second, directly incorporating external semantic priors into Transformer-based 3D architectures can be memory-intensive. Existing attempts to enhance 3D representations with multi-view image encoders or vision–language models (VLMs), including Contrastive Language–Image Pre-training (CLIP), typically rely on high-dimensional tokens and dense pairwise interactions, which rapidly exhaust GPU memory and restrict training to smaller windows or batch sizes (Dao et al., 2022; Chen et al., 2025; Yamazaki et al., 2024). These limitations call for a practical solution that incorporates VLM-derived semantic priors into sparse 3D representations while avoiding excessive memory overhead in semantic-geometric attention.

To address these challenges, we propose a CLIP-guided 3D segmentation framework that aligns vision–language semantic features with 3D point clouds and integrates them into sparse voxel tokens for memory-efficient window-based Transformer segmentation. Specifically, dense CLIP features extracted from multi-view RGB images are projected onto 3D points through visibility-aware alignment and view pooling, and are fused with relative geometric offsets and color cues to construct semantically aware sparse voxel tokens. Building on contextual relative signal encoding (cRSE) (Wu et al., 2021), we introduce a semantic-geometric extension that combines relative geometry, appearance variation, and a decoupled CLIP-induced discrepancy residual as a dynamic pairwise attention bias. To make this extension practical, block-wise online softmax computation is adapted to generate and consume bias tiles without materializing the full pairwise tensor.

Our contributions are threefold:

- We propose a CLIP-guided feature alignment framework that projects dense CLIP features from multi-view RGB images onto 3D points through visibility-aware alignment and view pooling. The aligned semantic features are fused with relative geometric offsets and color cues to construct semantically aware sparse voxel tokens for point-cloud segmentation.
- We extend contextual relative encoding with a decoupled CLIP-induced semantic residual and quantify its incremental contribution to the structural semantic–geometric attention bias within local windows.
- We adapt block-wise online softmax computation to generate and fuse cRSE bias tiles on the fly. This implementation avoids full pairwise bias materialization and reduces peak online training memory for the 3D stage by 25.7% at a window capacity of *N*_*w*_ = 32 on a single 24GB GPU.

## 2 Related work

### 2.1 3D scene segmentation

Early 3D scene segmentation methods primarily relied on point-based local aggregation [e.g., PointNet++ (Qi et al., 2017)], sparse convolutional operators, such as KPConv (Thomas et al., 2019) and SparseUNet (Choy et al., 2019; Gezawa et al., 2023), or efficient graph-convolutional architectures, such as MLGCN (Khodadad et al., 2024), to extract multi-scale geometric features from irregular 3D data. More recently, 3D Transformers have emerged as a powerful alternative for scalable context modeling (Wu et al., 2024; He et al., 2024; Lu and Zhang, 2026). To mitigate the quadratic complexity of global self-attention, partition-based or window-based Transformers (Lai et al., 2022; Wu et al., 2024; Yang et al., 2025b; Li et al., 2025) restrict attention to local or shifted spatial windows, offering a favorable trade-off between receptive field and computational efficiency. Despite their strong scalability, these architectures still rely predominantly on geometry-centric inductive biases (Zhao et al., 2021; Wu et al., 2022; Park et al., 2022), such as relative position encodings derived from spatial offsets. Although such inductive biases are effective for local geometry modeling, they provide limited guidance when spatially adjacent tokens correspond to different semantic categories, as frequently observed in cluttered scenes and long-tail settings. Existing window-based 3D Transformers mainly rely on geometric relative encoding to modulate local attention, while high-level semantic discrepancy is rarely converted explicitly into pairwise attention biases. To address this limitation, our semantic-geometric cRSE extension introduces aligned CLIP-induced discrepancy into local attention. It measures the discrepancy between neighboring voxel tokens in CLIP space and adds a decoupled residual to the structural attention bias.

### 2.2 3D vision–language learning

The strong open-vocabulary priors of vision–language models (VLMs) such as CLIP (Radford et al., 2021) have inspired a growing body of research on grounding 2D semantics in 3D scenes (Peng et al., 2023; Takmaz et al., 2023; Jatavallabhula et al., 2023; Chen et al., 2023; Yamazaki et al., 2024; Tai et al., 2026). Representative methods commonly transfer image-level, region-level, or dense 2D semantic features into 3D representations through multi-view correspondences. For example, OpenScene (Peng et al., 2023) projects 2D open-vocabulary features onto 3D points to enable open-vocabulary 3D scene understanding, while ConceptFusion (Jatavallabhula et al., 2023) fuses multi-view visual concepts into 3D maps for open-set scene representation. OpenMask3D (Takmaz et al., 2023) further exploits 2D foundation-model features for open-vocabulary 3D instance segmentation, and CLIP2Scene (Chen et al., 2023) investigates CLIP-guided 3D scene understanding by transferring 2D vision–language knowledge to point cloud representations. These methods demonstrate that projecting or aggregating 2D VLM features can effectively enrich point clouds with high-level semantic cues. Recent post-2024 studies have further explored vision–language priors for 3D scene understanding. MPEC (Wang Y. et al., 2025) introduces masked point–entity contrastive learning with 3D entity–language alignment and uses a frozen CLIP text encoder for 3D representation learning. PGOV3D (Zhang et al., 2025) develops a partial-to-global curriculum based on multi-view RGB-D observations, CLIP-related language supervision, a multimodal large language model, and a 2D segmentation foundation model. Open-Vocabulary Octree-Graph (Wang Z. et al., 2025) uses CLIP and OVSeg features to construct zero-shot open-vocabulary semantic and instance-level 3D scene representations. These recent studies confirm the continuing importance of vision–language features in 3D scene understanding, while covering different supervision and evaluation settings. While effective, most existing methods mainly use the projected CLIP features as additional point-level, voxel-level, or region-level descriptors, for example through feature concatenation, aggregation, or auxiliary semantic guidance (Wang Z. et al., 2025; Tai et al., 2026). MPEC further studies entity–language representation learning, whereas PGOV3D and Open-Vocabulary Octree-Graph primarily target open-vocabulary or zero-shot scene understanding. These settings differ from the closed-set supervised segmentation protocol considered in this work. Such designs enhance the semantic representation of 3D tokens, but they rarely use the semantic discrepancy between local tokens in the CLIP feature space to directly modulate attention computation. In contrast, our framework not only aligns multi-view CLIP features with 3D points through visibility-aware projection and view pooling, but also converts the aligned CLIP semantics into a dynamic pairwise bias for local attention. Specifically, we measure the semantic discrepancy between neighboring voxel tokens in the CLIP feature space and incorporate this discrepancy into cRSE as an additive bias to the attention logits. This design allows CLIP semantics to guide token-to-token attention weights within local windows, rather than serving only as static input features.

### 2.3 Memory-efficient attention

The memory bottleneck of self-attention has motivated extensive research on efficient attention mechanisms. In natural language processing (NLP) and 2D vision, hardware-aware methods such as FlashAttention (Dao et al., 2022; Dao, 2024) use I/O-aware block-wise tiling and streaming softmax computation to avoid materializing large intermediate attention matrices. Recent sparse or structured attention methods further improve scalability by restricting attention to selected tokens or local regions (Yuan et al., 2025). In 3D point cloud understanding, efficient Transformer designs commonly reduce attention cost through local windows, partition-based attention, voxelization, token reduction, or hardware-aware sparse computation (Wu et al., 2024; Chen et al., 2025; He et al., 2024). However, applying fused attention principles to sparse 3D architectures remains challenging. Point cloud windows usually contain irregular numbers of tokens, and sparse indexing introduces non-negligible gather/scatter overhead in window partitioning and token aggregation (Wu et al., 2024; Chen et al., 2025; He et al., 2024). Moreover, additional pairwise bias terms, such as relative positional encoding or semantic-geometric attention bias, are not directly supported by standard fused attention implementations (Dao et al., 2022; Dao, 2024; Yuan et al., 2025). In our cRSE attention, the bias for each token pair depends on relative geometry, appearance variation, and CLIP-induced semantic discrepancy. A naive implementation would pre-compute and store the full bias tensor of size *H*×*N*_*w*_×*N*_*w*_, resulting in $O\left(HN_{w}^{2}\right)$ auxiliary memory. To reduce this overhead, we use a block-wise streaming integration strategy. Instead of materializing the full cRSE bias tensor, we compute only the bias tile required by the current key/value block, add it directly to the attention logits, and discard it after updating the running softmax accumulation. This reduces cRSE bias storage from full pairwise tensor materialization to block-level intermediate buffers, enabling memory-efficient semantic-geometric attention for sparse 3D Transformers.

Table 1 summarizes the mechanism-level differences between our method and closely related approaches. Unlike methods that use CLIP features only as input-level descriptors or rely solely on structural relative encoding, our method introduces a decoupled pairwise semantic residual and integrates the resulting cRSE bias without materializing the full bias tensor.

**Table 1:** Mechanism-level comparison with closely related methods.

Method	Input-level CLIP	Pairwise sem. bias	Decoupled sem. branch	Full bias materialized	3D-stage cost reported
Swin3D (Yang et al., 2025b)	×	×	×	N/R	Partial
OpenScene (Peng et al., 2023)	✓	×	×	N/A	N/R
CLIP2Scene (Chen et al., 2023)	✓	×	×	N/A	N/R
Contextual relative position encoding (RPE) (Wu et al., 2021)	×	×	×	N/R	N/R
**Ours**	✓	✓	✓	×	✓

“N/R” denotes that the corresponding implementation or cost is not reported in the cited work; “N/A” denotes not applicable.

Boldface identifies the proposed method.

## 3 Methodology

Figure 1 summarizes the pipeline: frozen multi-view CLIP features are aligned with 3D points, embedded into sparse voxel tokens, and processed by a Swin3D-style encoder equipped with semantic-geometric cRSE. A block-wise implementation generates cRSE bias tiles only when they are consumed by attention.

**Figure 1:**
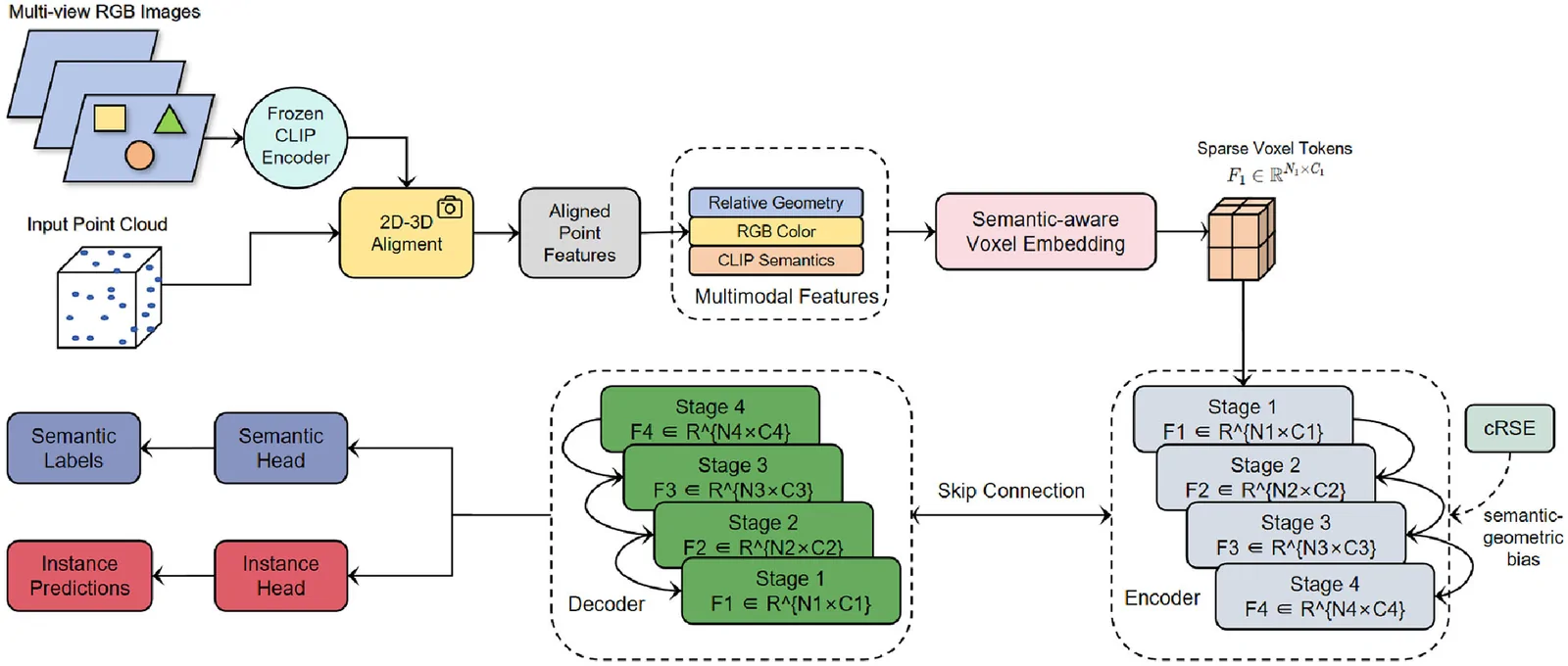
Overview of the proposed CLIP-guided sparse 3D segmentation framework.

### 3.1 Input representation and multimodal alignment

We align VLM-derived priors from multi-view images with 3D points before sparse voxel processing (Peng et al., 2023; Chen et al., 2023; Jatavallabhula et al., 2023; Wang Z. et al., 2025). Unlike point-level feature transfer alone, the aligned features are later reused to construct pairwise semantic attention biases.

#### 3.1.1 Voxelization and 2D semantic extraction

Given an input 3D point cloud with coordinates and colors, we denote the *i*-th point as (**x**_*i*_, **c**_*i*_), where ${\text{x}}_{i}={\left(x_{i},y_{i},z_{i}\right)}^{⊤}\in {\mathbb{R}}^{3}$ and ${\text{c}}_{i}={\left(r_{i},g_{i},b_{i}\right)}^{⊤}\in {\mathbb{R}}^{3}$. The point cloud is first discretized into a sparse voxel grid for subsequent sparse 3D processing (Choy et al., 2019; Graham et al., 2018). Meanwhile, the associated multi-view RGB images ${\left\{I^{\left(v\right)}\right\}}_{v=1}^{V}$ are fed into a frozen pre-trained CLIP image encoder Φ_CLIP_ to extract dense 2D semantic feature maps:


$${\text{F}}^{\left(v\right)}=\Phi_{\text{CLIP}}\left(I^{\left(v\right)}\right),\;{\text{F}}^{\left(v\right)}\in {\mathbb{R}}^{H^{\prime }\times W^{\prime }\times C_{\text{clip}}},$$

Here, *V* is the number of selected views, *v* is the camera-view index, *H*′ and *W*′ denote the height and width of the CLIP patch-feature map, respectively, namely the numbers of patch positions along its two spatial axes, and *C*_clip_ is the feature-channel dimension. In our implementation, *H*′ = *W*′ = 24 and *C*_clip_ = 1024. In practice, **F**^(*v*)^ is formed from the last-layer CLIP patch-token features excluding the global classification ([CLS]) token, reshaped into a 2D patch grid and normalized before feature sampling. These feature maps provide the 2D semantic cues that are subsequently projected and aligned with 3D points.

#### 3.1.2 Visibility-aware projection

To align 2D CLIP semantics with 3D points (Hu et al., 2021; Dai and Nießner, 2018), each 3D point is projected onto the image plane of a camera view *v*. Given the intrinsic matrix *K*^(*v*)^ and extrinsic matrix [*R*^(*v*)^|*t*^(*v*)^], the projected pixel coordinate of point **x**_*i*_ is computed as


$$\begin{array}{c} {\tilde{\text{u}}}_{i}^{\left(v\right)}\sim K^{\left(v\right)}\left[R^{\left(v\right)}|t^{\left(v\right)}\right]{\tilde{\text{x}}}_{i},\;{\text{u}}_{i}^{\left(v\right)}=\pi \left({\tilde{\text{u}}}_{i}^{\left(v\right)}\right), \end{array}$$ (1)

In Equation 1, *K*^(*v*)^∈ℝ^3 × 3^ is the camera intrinsic matrix, while *R*^(*v*)^∈ℝ^3 × 3^ and *t*^(*v*)^∈ℝ^3^ define the world-to-camera transformation. The vector ${\tilde{\text{x}}}_{i}={\left[{\text{x}}_{i}^{⊤},1\right]}^{⊤}$ is the homogeneous 3D coordinate, and ${\tilde{\text{u}}}_{i}^{\left(v\right)}$ is the homogeneous image coordinate. The symbol ~ denotes equality up to a nonzero scale, and π(·) performs perspective division. To suppress erroneous semantic alignment caused by occlusion or geometric inconsistency (Peng et al., 2023), we further apply a visibility-aware depth test. Let $d_{i}^{\left(v\right)}$ denote the projected depth of point **x**_*i*_ in view *v*, and let $D^{\left(v\right)}\left({\text{u}}_{i}^{\left(v\right)}\right)$ be the corresponding rendered or sensor depth. The visibility indicator is defined as


$$\begin{array}{c} m_{i}^{\left(v\right)}=1\left(|d_{i}^{\left(v\right)}-D^{\left(v\right)}\left({\text{u}}_{i}^{\left(v\right)}\right)|<\delta \right), \end{array}$$ (2)

Here, **1**(·) is the indicator function. The threshold δ = 0.05m is fixed for visibility filtering in all experiments and is not learned during training. Only visible projections with $m_{i}^{\left(v\right)}=1$ are used for CLIP feature sampling and subsequent view pooling.

#### 3.1.3 View pooling and aligned point representation

Since a 3D point may be visible in multiple views, we aggregate the corresponding CLIP features (Chen et al., 2023) through view pooling. Let Ω_img_ denote the image domain and define the valid-view set of point **x**_*i*_ as


$$V_{i}=\left\{v\in \left\{1,\ldots ,V\right\}:{\text{u}}_{i}^{\left(v\right)}\in \Omega_{\text{img}},\;m_{i}^{\left(v\right)}=1\right\}.$$

Let ${\text{f}}_{i}^{\left(v\right)}$ be the CLIP feature sampled at ${\text{u}}_{i}^{\left(v\right)}$ for $v\in V_{i}$. We compute the aligned CLIP semantic feature as


$$\begin{array}{c} {\text{s}}_{i}=\frac{\sum_{v\in V_{i}}w_{i}^{\left(v\right)}{\text{f}}_{i}^{\left(v\right)}}{\sum_{v\in V_{i}}w_{i}^{\left(v\right)}} \end{array}$$ (3)

where $w_{i}^{\left(v\right)}$ is the view-specific pooling weight. We define the point-level CLIP-validity flag as


$$\begin{array}{c} \rho_{i}^{\text{clip}}=1\left(|V_{i}|>0\right), \end{array}$$ (4)

In Equation 4, $\rho_{i}^{\text{clip}}=1$ indicates that point *i* receives at least one valid CLIP observation from the selected views. If $V_{i}=\emptyset$, we set **s**_*i*_ = **0** and record a separate invalidity flag, thereby avoiding an undefined zero denominator. In all experiments, we use uniform weights, namely $w_{i}^{\left(v\right)}=1$ for every valid projection, so that Equation 3 reduces to average pooling over the valid views of each point. The aligned point representation is subsequently combined with the intra-voxel coordinate offset, RGB color, and aligned CLIP feature CLIP_*f, i*_≡**s**_*i*_.

### 3.2 Semantically aware initial embedding

The aligned point features are converted into semantically aware sparse voxel tokens for the Swin3D-style backbone (Yang et al., 2025b).

#### 3.2.1 Fine-scale sparse voxelization and relative embedding

Let *v*_*s*_ be the voxel size, **ν**_*i*_ = ⌊**x**_*i*_/*v*_*s*_⌋ be the integer voxel coordinate obtained by element-wise flooring, ν(*i*) be the voxel index assigned to point *i*, and **c**_ν(*i*)_ be its center. The brackets in Equation 5 denote channel-wise concatenation. The input embedding retains intra-voxel geometry and concatenates it with color and CLIP semantics (Lai et al., 2022; Choy et al., 2019; Peng et al., 2023):


$$\begin{array}{c} {\text{e}}_{i}={\left[{\left({\text{x}}_{i}-{\text{c}}_{\nu \left(i\right)}\right)}^{⊤},{\text{c}}_{i}^{⊤},{\text{CLIP}}_{f,i}^{⊤}\right]}^{⊤}. \end{array}$$ (5)

Here **c**_*i*_ is the RGB vector. This embedding exposes geometry, appearance, and semantics from the first layer.

#### 3.2.2 Sparse convolutional encoding

Point embeddings in each non-empty voxel are average-pooled and passed through a 3 × 3 × 3 sparse convolution, batch normalization (BN), and a rectified linear unit (ReLU) (Graham et al., 2018; Choy et al., 2019). The resulting tensor ${\text{F}}_{1}\in {\mathbb{R}}^{N_{1}\times C_{1}}$ provides the initial sparse tokens. Here, *N*_1_ is the number of non-empty voxels, or equivalently the number of active sparse tokens, at the initial stage, and *C*_1_ is the corresponding feature-channel dimension. In our implementation, *C*_1_ = 48.

### 3.3 Semantic-geometric window attention via cRSE

Here, cRSE refers to contextual relative signal encoding, following the contextual relative-position formulation in (Wu et al. 2021). In each window, **p**_*i*_ is the center of voxel token *i* and **c**_*i*_ is its mean RGB color. The structural branch therefore uses token-level differences in geometry and appearance rather than raw point coordinates. Given the semantically aware initial sparse tensor ${\text{F}}_{1}\in {\mathbb{R}}^{N_{1}\times C_{1}}$ from the semantically aware initial embedding stage, we feed it into a hierarchical Swin3D-style backbone (Yang et al., 2025b) to learn multi-scale semantic-geometric representations. The backbone comprises four stages, where successive blocks alternate between window-based self-attention (W-MSA3D) and shifted-window self-attention (SW-MSA3D) (Liu et al., 2021) to enable efficient local context modeling and cross-window information exchange. However, standard 3D window attention mainly relies on geometric relative position encodings when modeling pairwise token relations, making it less effective in distinguishing physically adjacent regions with different semantics. We therefore extend this structural encoding (Wu et al., 2021) with a CLIP-induced semantic residual while retaining geometry and appearance cues.

#### 3.3.1 CLIP-induced semantic discrepancy

For voxel index ν, let


$$P_{\nu }=\left\{i:\nu \left(i\right)=\nu \right\}$$

denote the set of points assigned to voxel ν. We first count the points in this voxel that receive at least one valid CLIP observation:


$$\begin{array}{c} n_{\nu }^{\text{clip}}=\underset{i\in P_{\nu }}{\sum }\rho_{i}^{\text{clip}},\;m_{\nu }^{\text{clip}}=1\left(n_{\nu }^{\text{clip}}>0\right). \end{array}$$ (6)

The voxel-level CLIP feature is then obtained by averaging the valid point-level features:


$$g_{\nu }=\{\begin{array}{ll} \frac{1}{n_{\nu }^{\text{clip}}}\sum_{i\in P_{\nu }}\rho_{i}^{\text{clip}}s_{i}, & n_{\nu }^{\text{clip}}>0,\\ 0, & n_{\nu }^{\text{clip}}=0. \end{array}$$ (7)

Equations 6, 7 define the voxel-level CLIP-validity statistics and the pooled voxel-level CLIP feature, respectively. For a voxel token *i*, let ν_*i*_ denote the voxel represented by that token. The voxel-level validity flag $m_{\nu_{i}}^{\text{clip}}$ is distinct from the point–view visibility flag $m_{i}^{\left(v\right)}$ and the point-level aggregated flag $\rho_{i}^{\text{clip}}$. Specifically, $m_{\nu_{i}}^{\text{clip}}=1$ if at least one point assigned to voxel ν_*i*_ receives a valid CLIP observation from any selected view.

For tokens *i* and *j* in the same local window, the semantic discrepancy is defined as


$$\begin{array}{c} \delta_{ij}^{\text{sem}}=m_{\nu_{i}}^{\text{clip}}m_{\nu_{j}}^{\text{clip}}\left(1-\frac{{\text{g}}_{\nu_{i}}^{⊤}{\text{g}}_{\nu_{j}}}{\max\left(||{\text{g}}_{\nu_{i}}{||}_{2}||{\text{g}}_{\nu_{j}}{||}_{2},\epsilon \right)}\right). \end{array}$$ (8)

In Equation 8, ϵ = 10^−6^ is the numerical-stability constant used in all experiments. A larger value of $\delta_{ij}^{\text{sem}}$ indicates a stronger CLIP-space mismatch. The semantic discrepancy is set to zero whenever either represented voxel lacks a valid CLIP observation.

#### 3.3.2 Joint context and cRSE bias generation

To keep high-level semantics separate from low-level transformations, we form Δ**z**_*ij*_ = [**p**_*i*_−**p**_*j*_||**c**_*i*_−**c**_*j*_] and inject semantic discrepancy through a residual branch. The head-specific bias is


$$\begin{array}{c} B_{i,j,h}^{\text{cRSE}}={\text{w}}_{h}^{⊤}\left(t_{Q}\left(\Delta {\text{z}}_{ij}\right)+t_{K}\left(\Delta {\text{z}}_{ij}\right)\right)-\lambda_{s,h}\delta_{ij}^{\text{sem}},\;\lambda_{s,h}\geq 0, \end{array}$$ (9)

In Equation 9, || denotes channel-wise concatenation, *h*∈{1, …, *H*} indexes one of the *H* heads in the current attention block. The transformations *t*_*Q*_ and *t*_*K*_ map Δ**z**_*ij*_ to a shared latent dimension, **w**_*h*_ projects this representation to a scalar bias for head *h*, and λ_*s, h*_ is a non-negative head-specific semantic coefficient implemented with a softplus parameterization. The negative residual suppresses attention between semantically inconsistent neighbors without entangling CLIP features with structural transformations.

#### 3.3.3 Integration into window attention

The bias is added directly to window attention:


$$\begin{array}{c} {\text{o}}_{i,h}=\underset{j}{\sum }{\mathrm{softmax}}_{j}\left(\frac{{\text{q}}_{i,h}^{⊤}{\text{k}}_{j,h}}{\sqrt{d}}+B_{i,j,h}^{\text{cRSE}}\right){\text{v}}_{j,h}. \end{array}$$ (10)

In Equation 10, **q**_*i, h*_, **k**_*j, h*_, and **v**_*j, h*_ are the query, key, and value vectors for tokens *i* and *j* in the current local window. They are obtained as ${\text{q}}_{i,h}={\text{F}}_{i}W_{h}^{Q}$, ${\text{k}}_{j,h}={\text{F}}_{j}W_{h}^{K}$, and ${\text{v}}_{j,h}={\text{F}}_{j}W_{h}^{V}$, where $W_{h}^{Q}$, $W_{h}^{K}$, and $W_{h}^{V}$ are learned head-specific projection matrices. The dimension *d* is the per-head feature dimension, the index *j* ranges over valid key/value tokens in the same window, and softmax_*j*_ normalizes the logits over those tokens.

Figure 2 illustrates the complete cRSE computation and streaming-attention process. The original voxel tokens generate the query, key, and value features, while relative geometry, color difference, and CLIP-space are the structural and semantic branches that process relative and semantic discrepancy to construct the attention bias. This bias is generated and consumed tile by tile during block-wise attention.

**Figure 2:**
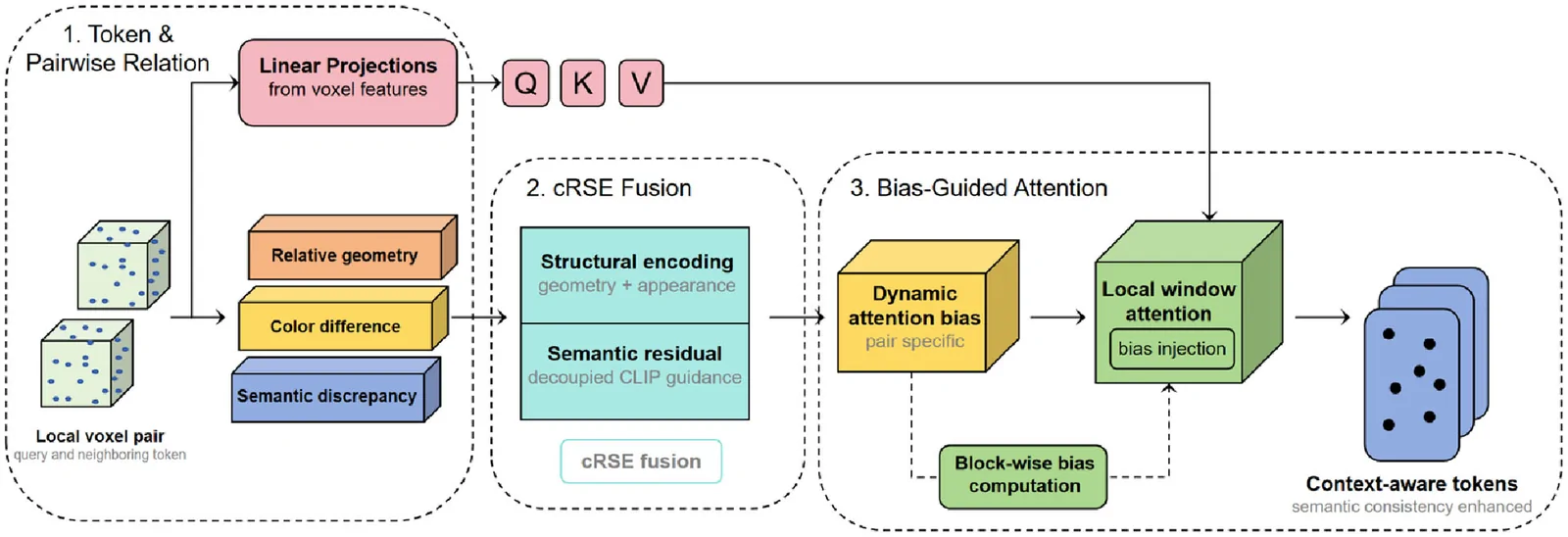
Semantic-geometric cRSE and its streaming integration. Geometry and color form the structural bias, while CLIP discrepancy is injected through a decoupled semantic residual; bias tiles are generated and consumed on the fly during block-wise attention.

### 3.4 Memory-efficient streaming integration for cRSE

Naively storing $B^{\text{cRSE}}\in {\mathbb{R}}^{H\times N_{w}\times N_{w}}$ incurs $O\left(HN_{w}^{2}\right)$ auxiliary memory. We instead partition keys and values into tiles. Let *N*_*w*_ be the window capacity and let the keys and values be partitioned into *T* tiles. For a fixed query, tile *t* produces $\alpha_{t}=\text{q}{\text{K}}_{t}^{⊤}/\sqrt{d}+{\text{B}}_{t}^{\text{cRSE}}$, where **K**_*t*_, **V**_*t*_, and ${\text{B}}_{t}^{\text{cRSE}}$ are the key, value, and cRSE-bias tiles. The index *j* runs over entries in tile *t*. The variables *m*_*t*_, ℓ_*t*_, and **a**_*t*_ denote the running maximum, softmax normalization factor, and value accumulator after tile *t*. We initialize *m*_0_ = −∞, ℓ_0_ = 0, and **a**_0_ = **0**; padding and shifted-window masks are applied to **α**_*t*_ before the update. Equation 11 updates the running maximum, normalization factor, and value accumulator tile by tile.


$$\begin{array}{l} m_{t}=\max\left(m_{t-1},\max\alpha_{t}\right),\\ \ell_{t}=e^{m_{t-1}-m_{t}}\ell_{t-1}+\underset{j}{\sum }e^{\alpha_{t,j}-m_{t}},\\ {\text{a}}_{t}=e^{m_{t-1}-m_{t}}{\text{a}}_{t-1}+\underset{j}{\sum }e^{\alpha_{t,j}-m_{t}}{\text{v}}_{t,j},\;\text{o}={\text{a}}_{T}/\ell_{T}. \end{array}$$ (11)

Here, α_*t, j*_ and **v**_*t, j*_ are the *j*-th logit and value vector in tile *t*, while **o** is the final attention output for the query. The required cRSE tile is generated together with each dot-product tile, consumed by this recurrence, and discarded. Thus, the forward computation retains block-level bias buffers instead of the full pairwise bias tensor. The reported memory measurements cover the online 3D segmentation stage with fixed, pre-computed CLIP features; they do not include offline CLIP extraction, virtual-view rendering, alignment, or cache storage.

### 3.5 Multi-scale decoding and segmentation heads

U-Net-style sparse decoder mirrors the four encoder stages, using transposed sparse convolutions and skip connections to restore voxel resolution (Ronneberger et al., 2015; Choy et al., 2019). The semantic head predicts per-voxel class logits, which are mapped to raw points by the deterministic point-to-voxel correspondence. A lightweight instance branch predicts center offsets, embeddings, and objectness; at inference, objectness-filtered points are clustered jointly by shifted coordinates and embeddings, followed by semantic voting and mask-level non-maximum suppression (NMS). These standard heads are retained only to evaluate whether the proposed representation transfers to both semantic and instance segmentation.

The semantic loss combines cross-entropy and Lovász–Softmax (Berman et al., 2018). The instance loss combines offset regression, objectness binary cross-entropy (BCE), and discriminative embedding losses. The complete objective is


$$\begin{array}{c} L=\underset{L_{\text{sem}}}{\underset{︸}{L_{\text{CE}}+L_{\text{Lovasz}}}}+\lambda_{\text{ins}}\underset{L_{\text{ins}}}{\underset{︸}{\left(L_{\text{off}}+\lambda_{\text{obj}}L_{\text{obj}}+\lambda_{\text{emb}}L_{\text{emb}}\right)}}. \end{array}$$ (12)

In Equation 12, $L_{\text{CE}}$ and $L_{\text{Lovasz}}$ are the semantic cross-entropy and Lovász–Softmax losses. The terms $L_{\text{off}}$, $L_{\text{obj}}$, and $L_{\text{emb}}$ denote the offset-regression, objectness, and discriminative-embedding losses, respectively. The coefficients are fixed to λ_ins_ = 1.0, λ_obj_ = 1.0, and λ_emb_ = 1.0 in all experiments. Definitions of the standard instance losses, point grouping procedure, and main inference thresholds are provided in the Supplementary material.

## 4 Experiments

### 4.1 Experimental setup

#### 4.1.1 Datasets and protocols

We evaluate on ScanNet v2 (Dai et al., 2017), ScanNet200 (Rozenberszki et al., 2022), and the Stanford Large-Scale 3D Indoor Spaces (S3DIS) dataset (Armeni et al., 2016). ScanNet v2 contains 1,201 training scans and 312 validation scans, together with 100 held-out test scans used by the official benchmark. We train on the official training split and report results on the 312-scan validation split; the hidden test set is not used. For ScanNet, semantic and instance segmentation are evaluated over 20 and 18 categories, respectively. ScanNet200 uses the same 1,201/312/100 training–validation–test partition and extends the label space to 200 semantic and 200 instance categories, which are grouped into head, common, and tail subsets according to occurrence frequency. The 100-scan hidden test split is not used, and all ScanNet200 results are reported on the validation split. S3DIS contains six areas, 271 rooms, and 13 annotated categories. Under the Area-5 protocol, the 203 rooms from Areas 1, 2, 3, 4, and 6 are used for training, whereas the 68 rooms in Area 5 are used exclusively for evaluation; no separate S3DIS validation split is used.

#### 4.1.2 Evaluation metrics

For semantic segmentation, the intersection-over-union for class *c* and its class average are


$$\begin{array}{c} {\text{IoU}}_{c}=\frac{{\text{TP}}_{c}}{{\text{TP}}_{c}+{\text{FP}}_{c}+{\text{FN}}_{c}},\;\text{mIoU}=\frac{1}{C}\overset{C}{\underset{c=1}{\sum }}{\text{IoU}}_{c}, \end{array}$$ (13)

In Equation 13, TP_*c*_, FP_*c*_, and FN_*c*_ denote the numbers of true-positive, false-positive, and false-negative points for class *c*, respectively, and *C* is the number of evaluated semantic classes. For instance segmentation, mask IoU is the intersection-over-union between a predicted and a ground-truth point mask. A prediction is a true positive when it has the correct category, reaches mask IoU ≥0.5 with an unmatched ground-truth instance, and is selected by one-to-one score-ordered matching. Let *r*∈[0, 1] denote recall and let *p*_*c*_(*r*) denote the interpolated precision of class *c* at recall *r*. We report


$$\begin{array}{c} {\text{AP}}_{50}^{\left(c\right)}=\int_{0}^{1}p_{c}\left(r\right)\text{d}r,\;{\text{mAP}}_{50}=\frac{1}{C_{\text{ins}}}\overset{C_{\text{ins}}}{\underset{c=1}{\sum }}{\text{AP}}_{50}^{\left(c\right)}, \end{array}$$ (14)

In Equation 14, *C*_ins_ is the number of valid instance categories. Head/common/tail AP_50_ values are the same class-wise average restricted to each ScanNet200 frequency group.

#### 4.1.3 Multimodal input protocol

ScanNet provides approximately 2.5 million posed RGB-D frames with calibrated camera intrinsics, camera poses, and depth maps, and ScanNet200 reuses the same RGB-D sequences. For each ScanNet or ScanNet200 scene, frames with invalid poses or missing depth maps are removed. The remaining frames are ordered by temporal index, and 32 approximately uniformly spaced frames are selected using *i*_*k*_ = round(*k*(*M*−1)/31), where *k* = 0, …, 31 and *M* is the number of valid frames. The resulting view list is fixed across all experiments. S3DIS provides colored point clouds but does not provide posed RGB-D video sequences in the same form as ScanNet. We therefore deterministically generate virtual RGB-D views from the normalized room bounding box using eight azimuth directions, four normalized camera heights, fixed camera intrinsics, and z-buffer rendering. Each camera is oriented toward the room center. Exactly 32 deterministic candidate views are generated for each S3DIS room using the fixed camera layout described in the Supplementary material. Candidate views with projected-point coverage below τ_cov_ = 0.05 are excluded from feature alignment. Consequently, each room uses a deterministic valid subset of up to 32 views; the exact number depends on the room-specific coverage test, and rejected views are not replaced. The remaining valid views are used for CLIP feature extraction and 2D–3D alignment. We freeze the CLIP ViT-L/14 encoder and resize each RGB image to 336 × 336. The normalized last-layer patch tokens form a 24 × 24 × 1, 024 feature map. Features are bilinearly sampled, retained when the depth discrepancy is below 0.05 m, and uniformly averaged across valid views. Points without valid observations receive a zero feature and a separate validity mask. Point-aligned CLIP features are cached in half precision and used during both 3D training and inference. Detailed camera parameters and the complete pipeline are provided in the Supplementary material.

#### 4.1.4 Implementation details

The 3D network is implemented in PyTorch/OpenMMLab and trained on one 24GB RTX 4090. We use AdamW with learning rate 10^−3^, weight decay 5 × 10^−2^, cosine decay, 1500 warmup iterations, batch size 4, voxel size *v*_*s*_ = 0.02 m, and base window size *N*_*w*_ = 32. We apply standard geometric and color augmentations. All controlled variants share the same training schedule, instance head, and post-processing settings. Unless otherwise stated, the main experiments use seed 42. The sequential component comparison in Table 2 is reported from a separately executed single-run diagnostic, whereas Table 3 reports an independent repeated-seed evaluation using seeds 41, 42, and 43. Results from these two experiment groups are therefore not treated as the same training runs. The Supplementary material provides the deterministic virtual-camera configuration and the complete ordered protocol for view generation, CLIP extraction, 2D–3D alignment, feature caching, 3D training, inference, and profiling.

**Table 2:** Protocol-matched cross-task component comparison.

Variant	Swin3D backbone	Prediction head	CLIP input	Geometry–color cRSE	Semantic residual	ScanNet mIoU	ScanNet200 AP_50_
Swin3D (reported)	✓	*Original*	×	×	×	74.5	23.5
Swin3D (retrained)	✓	*Ours*	×	×	×	73.30	28.66
+ CLIP input	✓	*Ours*	✓	×	×	76.12	35.80
+ geometry–color cRSE	✓	*Ours*	✓	✓	×	76.48	39.94
**Full model**	✓	*Ours*	✓	✓	✓	**76.82**	**40.86**

The first row reports the published Swin3D benchmark result. The remaining rows use the same backbone, prediction head, data split, training schedule, and post-processing, with the listed components added successively. The table reports ScanNet semantic mIoU and ScanNet200 instance AP_50_.

Bold values indicate the best result within the corresponding dataset/task group or controlled comparison.

**Table 3:** Stability of key configurations across three independent random seeds.

Dataset/task	Variant	Seed 41	Seed 42	Seed 43	Mean ±std
ScanNet semantic	3D-only baseline	73.3	73.5	73.4	73.40 ± 0.10
	CLIP input only	75.8	76.1	76.0	75.97 ± 0.15
	CLIP input + geometry–color cRSE	76.2	76.5	76.3	76.33 ± 0.15
	**Full model**	76.5	76.8	76.7	**76.67** **±0.15**
ScanNet200 instance	3D-only baseline	28.1	28.5	28.6	28.40 ± 0.26
	+ CLIP input	35.3	35.7	35.8	35.60 ± 0.26
	**Full model**	40.4	40.8	40.9	**40.70** **±0.26**

Mean ± std is reported, with the standard deviation computed as the sample standard deviation over seeds 41, 42, and 43.

Bold values indicate the best result within the corresponding dataset/task group or controlled comparison.

### 4.2 Comparison with state-of-the-art methods

We report both published benchmark results and controlled retrained variants. The retrained variants use the same backbone, prediction head, data split, training schedule, and post-processing, and provide the component-wise comparison of the CLIP input, geometry–color cRSE, and semantic residual.

#### 4.2.1 Protocol-matched controlled comparison

Table 2 presents a protocol-matched cross-task component comparison. Starting from the retrained Swin3D configuration, the CLIP input, geometry–color cRSE, and semantic residual are added successively while keeping the backbone, prediction head, data split, training schedule, and post-processing fixed. The table reports both ScanNet semantic mIoU and ScanNet200 instance AP_50_, providing a common cumulative view of the component contributions across two segmentation tasks.

On ScanNet, mIoU increases from 73.30 for the retrained Swin3D configuration to 76.12 with CLIP input, 76.48 with geometry–color cRSE, and 76.82 for the full model. Thus, the main performance increase is associated with the aligned CLIP input, while geometry–color cRSE and the semantic residual provide smaller incremental gains of 0.36 and 0.34 mIoU points, respectively. On ScanNet200, the corresponding AP_50_ values are 28.66, 35.80, 39.94, and 40.86. The semantic residual adds 0.92 AP_50_ points after the CLIP input and geometry–color cRSE have already been enabled. A finer branch-level analysis on ScanNet is provided separately in Table 4.

**Table 4:** Fine-grained branch-level ablations on the ScanNet validation split (mIoU, %).

Variant	CLIP input	Geometry	Color	Semantic residual	mIoU
3D-only baseline^†^					73.4
Geometry cRSE		✓			74.6
Geometry–color cRSE		✓	✓		75.1
CLIP input only^†^	✓				76.0
CLIP input + geometry–color cRSE^†^	✓	✓	✓		76.3
CLIP input + geometry cRSE + semantic residual	✓	✓		✓	76.3
**Full model** ^†^	✓	✓	✓	✓	**76.7**

The geometry, color, and semantic-residual columns indicate the branches included in the cRSE formulation. † denotes the mean over seeds 41, 42, and 43; the remaining diagnostic variants use seed 42.

Bold values indicate the best result within the corresponding dataset/task group or controlled comparison.

#### 4.2.2 Published benchmark comparisons

Tables 5, 6 compare our results with selected published benchmarks under their reported evaluation settings. To broaden the comparison with recent 3D methods using CLIP or related vision–language models, we include MPEC in the benchmark tables and summarize the reported results of PGOV3D and Open-Vocabulary Octree-Graph in Table 7.

**Table 5:** Semantic segmentation results on ScanNet, ScanNet200, and S3DIS.

Dataset	Task	Method	Supervision	Input/pretraining	Split	Metric	Result	Source
ScanNet	Semantic	Swin3D	Closed-set supervised	XYZ + RGB/as reported	Official val	mIoU	74.5	Yang et al., 2025b
ScanNet	Semantic	MPEC	Fine-tuned downstream	XYZ + RGB/MPEC 3D-VL pretraining	Official val	mIoU	75.8	Wang Y. et al., 2025
ScanNet	Semantic	Ours (3D-only)	Closed-set supervised	XYZ + RGB/from scratch	Official val	mIoU	73.40 ± 0.10	Our work (3 seeds)
ScanNet	Semantic	Ours (full)	Closed-set supervised	XYZ + RGB + CLIP^*^/frozen CLIP	Official val	mIoU	**76.67 ± 0.15**	Our work (3 seeds)
ScanNet200	Semantic	Swin3D	Closed-set supervised	XYZ + RGB/as reported	Official val	mIoU	30.2	Yang et al., 2025b
ScanNet200	Semantic	MPEC	Fine-tuned downstream	XYZ + RGB/MPEC 3D-VL pretraining	Official val	mIoU	31.8	Wang Y. et al., 2025
ScanNet200	Semantic	Ours (full)	Closed-set supervised	XYZ + RGB + CLIP^*^/frozen CLIP	Official val	mIoU	**33.2**	Our work (single run)
S3DIS	Semantic	Swin3D	Closed-set supervised	XYZ + RGB/as reported	Area-5	mIoU	72.0	Yang et al., 2025b
S3DIS	Semantic	Ours (full)	Closed-set supervised	XYZ + RGB + CLIP^*^/frozen CLIP	Area-5	mIoU	**73.0**	Our work (single run)

Published results are quoted from the cited papers, while our results are evaluated on the stated splits. The MPEC rows report its published downstream fine-tuning results after 3D vision–language pretraining. CLIP^*^ denotes frozen CLIP features aligned from native RGB-D views for ScanNet and ScanNet200, and from rendered virtual RGB-D views for S3DIS.

Bold values indicate the best result within the corresponding dataset/task group or controlled comparison.

**Table 6:** Instance segmentation results on ScanNet, ScanNet200, and S3DIS.

Dataset	Task	Method	Supervision	Input/pretraining	Split	Metric	Result	Source
ScanNet	Instance	Swin3D	Closed-set supervised	XYZ + RGB/as reported	Official val	AP_50_	69.4	Yang et al., 2025b
ScanNet	Instance	MPEC	Fine-tuned downstream	XYZ + RGB/MPEC 3D-VL pretraining	Official val	AP_50_	62.5	Wang Y. et al., 2025
ScanNet	Instance	Mask3D	Closed-set supervised	XYZ + RGB/as reported	Official val	AP_50_	**78.0**	Schult et al., 2023
ScanNet	Instance	Ours (full)	Closed-set supervised	XYZ + RGB + CLIP^*^/frozen CLIP	Official val	AP_50_	76.9	Our work (single run)
ScanNet200	Instance	Swin3D	Closed-set supervised	XYZ + RGB/as reported	Official val	AP_50_	23.5	Yang et al., 2025b
ScanNet200	Instance	MPEC	Fine-tuned downstream	XYZ + RGB/MPEC 3D-VL pretraining	Official val	AP_50_	31.6	Wang Y. et al., 2025
ScanNet200	Instance	Mask3D	Closed-set supervised	XYZ + RGB/as reported	Official val	AP_50_	38.8	Schult et al., 2023
ScanNet200	Instance	Ours (full)	Closed-set supervised	XYZ + RGB + CLIP^*^/frozen CLIP	Official val	AP_50_	**40.70 ± 0.26**	Our work (3 seeds)
S3DIS	Instance	PointGroup	Closed-set supervised	XYZ + RGB/as reported	Area-5	AP_50_	57.8	Jiang et al., 2020
S3DIS	Instance	Swin3D	Closed-set supervised	XYZ + RGB/as reported	Area-5	AP_50_	65.4	Yang et al., 2025b
S3DIS	Instance	Ours (full)	Closed-set supervised	XYZ + RGB + CLIP^*^/frozen CLIP	Area-5	AP_50_	**69.6**	Our work (single run)

Published results are quoted from the cited papers, while our results are evaluated on the stated splits. The MPEC rows report its published downstream fine-tuning results, originally given as mAP@0.5 and denoted as AP_50_ here for consistency. The PointGroup result on S3DIS follows the Area-5 protocol. CLIP^*^ denotes frozen CLIP features aligned from native RGB-D views for ScanNet and ScanNet200, and from rendered virtual RGB-D views for S3DIS.

Bold values indicate the best result within the corresponding dataset/task group or controlled comparison.

**Table 7:** Recent post-2024 CLIP/VLM-based 3D segmentation results together with our full model.

Task	Method	Setting	ScanNet	ScanNet200	S3DIS	Source
Semantic	MPEC	Fine-tuned downstream	75.8	31.8	–	Wang Y. et al., 2025
Semantic	PGOV3D	Open-vocabulary/zero-shot	59.5	9.3	43.2	Zhang et al., 2025
Semantic	Open-Vocabulary Octree-Graph	Zero-shot, CLIP ViT-H-14	35.6^†^	–	–	Wang Z. et al., 2025
**Semantic**	**Ours (full)**	**Closed-set supervised**	**76.67 ± 0.15**	**33.2**	**73.0**	**Our work**
Instance	MPEC	Fine-tuned downstream	62.5	31.6	–	Wang Y. et al., 2025
Instance	Open-Vocabulary Octree-Graph	Zero-shot	–	25.8^‡^	–	Wang Z. et al., 2025
**Instance**	**Ours (full)**	**Closed-set supervised**	**76.9**	**40.70 ± 0.26**	**69.6**	**Our work**

The reported values follow the evaluation settings of the corresponding methods. MPEC reports downstream fine-tuning results, while PGOV3D and Open-Vocabulary Octree-Graph report open-vocabulary or zero-shot results.

^†^ Open-Vocabulary Octree-Graph also reports 39.3 ScanNet mIoU when using an OVSeg backbone; the value shown here corresponds to its CLIP ViT-H-14 backbone.

^‡^ The ScanNet200 instance result is reported as AP_50_ under the zero-shot setting. MPEC reports instance performance as mAP@0.5, denoted as AP_50_ here for consistency.

Bold values indicate the results of our full model.

The mean and standard deviation reported for ScanNet are obtained from the repeated-seed evaluation. The single-run results in Table 2 come from the separate controlled component experiment and are used to measure the incremental contribution of each component.

For instance segmentation, the full model achieves an AP_50_ of 76.9% on ScanNet, 40.70 ± 0.26 on ScanNet200 across three seeds, and 69.6% on S3DIS Area 5. The corresponding comparisons with published methods are reported in Table 6.

Our full model achieves an AP_50_ of 76.9 on ScanNet, 40.70 ± 0.26 on ScanNet200 over three seeds, and 69.6 on S3DIS Area 5. On ScanNet200 and S3DIS, these results exceed the published values listed in the table, whereas Mask3D obtains the highest ScanNet AP_50_. The incremental contributions of the CLIP input, geometry–color cRSE, and semantic residual are examined separately in Table 2.

#### 4.2.3 Recent CLIP/VLM-based contextual comparisons

Recent CLIP/VLM-based methods published after 2024, together with our full model, are summarized in Table 7. The reported results are listed with their corresponding tasks and evaluation settings.

#### 4.2.4 Broader literature reference

Table 8 summarizes additional representative published point-cloud segmentation results on ScanNet, ScanNet200, and S3DIS. Recent CLIP/VLM-based results are reported separately in Table 7. Semantic-segmentation results in this table are reported using mIoU, whereas instance-segmentation results are reported using AP_50_. For methods whose official implementations or complete experimental configurations were unavailable, we report the values from the corresponding publications rather than reproducing those methods under our experimental setup.

**Table 8:** Representative published results and results for our full model.

Task	Method	ScanNet	ScanNet200	S3DIS	Source
Semantic	MinkowskiNet	72.2	–	65.4	Choy et al., 2019
Semantic	PointTransformer	70.6	–	70.4	Zhao et al., 2021
Semantic	PointNeXt	71.5	–	70.5	Qian et al., 2022
Semantic	Stratified Transformer	73.7	–	72.0	Lai et al., 2022
Semantic	Point Mamba	75.7	–	–	Liang et al., 2024
Semantic	OA-CNNs	76.1	32.3	71.1	Peng et al., 2024
Semantic	OctFormer	76.6	32.6	–	Wang, 2023
Semantic	**Ours (full)**	**76.7**	**33.2**	**73.0**	**Our work**
Instance	PointGroup	63.6	–	57.8	Jiang et al., 2020
Instance	HAIS	69.9	–	–	Chen et al., 2021
Instance	MinkowskiNet	61.5	15.2	56.8	Choy et al., 2019
Instance	PointNeXt	64.2	–	60.6	Qian et al., 2022
Instance	Swin3D	69.4	23.5	65.4	Yang et al., 2025b
Instance	Mask3D	**78.0**	38.8	–	Schult et al., 2023
Instance	**Ours (full)**	76.9	**40.7**	**69.6**	**Our work**

Semantic-segmentation results are reported as mIoU, and instance-segmentation results are AP_50_. The results of our method use frozen CLIP features aligned from native RGB-D views for ScanNet and ScanNet200, and from rendered virtual RGB-D views for S3DIS.

Bold values indicate the best result within the corresponding dataset/task group or controlled comparison.

### 4.3 Ablation studies

We first evaluate the proposed components on the ScanNet validation split under the same training and evaluation settings. The largest improvement is obtained by adding the aligned CLIP input. Geometry–color cRSE provides a further gain, while the semantic residual improves mIoU by an additional 0.3–0.4 points in the reported experiments. These results indicate that the semantic residual acts as a complementary refinement of the semantic–geometric attention bias. For variants marked with †, we report the mean over seeds 41, 42, and 43, rounded to one decimal; the remaining diagnostic variants are evaluated with seed 42.

The cross-task cumulative comparison is given in Table 2. A finer branch-level analysis of the ScanNet semantic task is provided in Table 4, while alternative semantic-residual formulations are examined in Table 9.

**Table 9:** Semantic-bias formulation on the ScanNet validation split.

Pairwise semantic design	Decoupled	Extra params	Peak memory (GB)	Step time (ms)	mIoU
None (CLIP input + structural cRSE)	N/A	0	13.2	447	76.45
Cosine discrepancy, joint fusion	×	0	13.4	456	76.48
ℓ_2_ distance residual	✓	0	13.5	457	76.61
Two-layer MLP bias	✓	0.13 M	13.8	473	76.75
**Cosine discrepancy residual (ours)**	✓	*H* scalars	13.5	460	**76.82**

Memory and step time are measured on one RTX 4090 with batch size 4 and *N*_*w*_ = 32.

Bold values indicate the best result within the corresponding dataset/task group or controlled comparison.

#### 4.3.1 Statistical stability across random seeds

To assess the stability of the observed improvements, we independently retrain the key configurations with seeds 41, 42, and 43. Table 3 reports the results for each run together with the sample mean and standard deviation. On ScanNet, the mean mIoU increases from 73.40 for the 3D-only baseline to 75.97 with CLIP input, 76.33 with geometry–color cRSE, and 76.67 for the full model. The full model exceeds the geometry–color cRSE configuration by 0.3, 0.3, and 0.4 points for the three seeds, respectively. On ScanNet200, the mean instance AP_50_ increases from 28.40 for the 3D-only configuration with our instance head to 35.60 with CLIP input and to 40.70 for the full model, showing consistent gains across random seeds.

#### 4.3.2 Semantic-bias formulation

Table 9 compares pairwise CLIP-discrepancy formulations while keeping the CLIP input, structural cRSE, backbone, prediction head, and training settings fixed. The CLIP-input-plus-structural-cRSE configuration serves as the reference and obtains 76.45 mIoU in this formulation study. Directly coupling the cosine discrepancy with the structural branch provides only a marginal gain, whereas decoupled ℓ_2_ and two-layer MLP residuals reach 76.61 and 76.75 mIoU, respectively. The proposed decoupled cosine residual reaches 76.82 mIoU, corresponding to a 0.37-point improvement over the structural reference. Compared with the two-layer MLP residual, it reduces peak memory by 0.3 GB and step time by 13 ms while improving mIoU by 0.07 points. These single-run results motivate the selected formulation, while the small differences among the strongest variants should be interpreted cautiously.

#### 4.3.3 Hyperparameter sensitivity

The semantic residual also depends on how its coefficient λ_*s, h*_ is constrained and shared. Table 10a compares fixed, unconstrained, and softplus-parameterized variants while keeping the semantic-bias formulation unchanged. Fixed coefficients of 0.25 and 0.50 obtain 76.57 and 76.67 mIoU, respectively. Without a non-negativity constraint, the learned scalar becomes −0.08, reversing the intended suppression of semantically inconsistent neighbors and reducing mIoU to 76.51. A non-negative learned scalar improves the result to 76.76. The proposed per-head softplus parameterization reaches 76.82 mIoU, with learned coefficients ranging from 0.18 to 0.71 across heads. This indicates that different heads can assign different weights to semantic disagreement. The 0.06-point gain over the learned scalar is modest, while the per-head parameterization provides greater flexibility.

Table 10Hyperparameter sensitivity on the ScanNet validation split.

**Table:** 

λ_*s, h*_ design	Non-negative	Per head	Learned value/ range	mIoU
(a) Semantic-weight parameterization				
Fixed scalar 0.25	✓	×	0.25	76.57
Fixed scalar 0.50	✓	×	0.50	76.67
Unconstrained learned scalar	×	×	−0.08	76.51
Softplus learned scalar	✓	×	0.43	76.76
**Softplus learned per-head (ours)**	✓	✓	0.18–0.71	**76.82**

**Table:** 

**δ (m)**	**Valid points (%)**	**mIoU**
(b) Depth-consistency threshold		
0.02	87.4	74.94
0.05	89.7	**76.82**
0.10	89.9	75.15

(a) Comparison of semantic-weight parameterizations; the coefficient range is the observed minimum–maximum across learned head-specific coefficients. (b) Sensitivity to the depth-consistency threshold used for visibility-aware CLIP alignment. The ‘Valid points' column denotes the percentage of points receiving at least one valid CLIP observation. All threshold variants use uniform view pooling and otherwise identical experimental settings.

Bold values indicate the best result within the corresponding dataset/task group or controlled comparison.

We further evaluate the sensitivity to the depth-consistency threshold δ in Equation 2, which determines whether a projected observation is retained during visibility-aware CLIP alignment. Table 10b reports the valid-point coverage and ScanNet validation mIoU for three threshold values, with uniform view pooling and all other experimental settings held fixed. Reducing δ from 0.05m to 0.02m decreases the percentage of points receiving at least one valid CLIP observation from 89.7% to 87.4% and reduces mIoU from 76.82 to 74.94. Increasing δ to 0.10m slightly increases the valid-point coverage to 89.9%, but reduces mIoU to 75.15. These results indicate that a stricter threshold removes useful observations, whereas a looser threshold can retain less reliable 2D–3D correspondences. We therefore use δ = 0.05m in all reported experiments as a balance between semantic-feature coverage and alignment reliability.

#### 4.3.4 Instance-level and long-tail analysis on ScanNet200

The ScanNet and ScanNet200 ablations examine complementary aspects of the proposed framework rather than repeating the same experiment. Table 4 evaluates semantic segmentation on ScanNet using mIoU and separates the geometry, color, and semantic-residual branches. In contrast, the following ScanNet200 study evaluates instance segmentation using overall and group-wise Head/Common/Tail AP_50_ under a larger and more imbalanced category space. We analyze instance segmentation across the Head, Common, and Tail groups of ScanNet200. Table 11 reports overall and group-wise AP_50_ for the controlled configurations. Overall AP_50_ increases from 28.4 for the 3D-only configuration to 35.6 with CLIP input, 39.8 with geometry–color cRSE, and 40.7 for the full model. Tail AP_50_ increases from 28.1 to 34.3, 39.7, and 40.2, respectively. Figure 3 compares the published Swin3D result with the full model, showing a 20.7-point Tail AP_50_ difference, from 19.5 to 40.2. This difference reflects the complete system rather than the semantic residual alone. Within the controlled chain, adding the semantic residual improves Tail AP_50_ by 0.5 points, from 39.7 to 40.2, after CLIP input and geometry–color cRSE have been enabled.

**Table 11:** Instance-level ablations on the ScanNet200 validation split (AP_50_).

Variant	Our head	CLIP input	Geometry–color cRSE	Semantic residual	AP_50_	Head	Common	Tail
Published Swin3D					23.5	26.5	24.1	19.5
3D-only configuration	✓				28.4	30.5	26.6	28.1
+ CLIP input	✓	✓			35.6	37.3	35.2	34.3
+ geometry–color cRSE	✓	✓	✓		39.8	42.9	36.8	39.7
**Full model**	✓	✓	✓	✓	**40.7**	**44.5**	**37.4**	**40.2**

Overall, Head, Common, and Tail scores are reported for each controlled configuration. Blank entries indicate components that are not used or are not applicable.

Bold values indicate the best result within the corresponding dataset/task group or controlled comparison.

**Figure 3:**
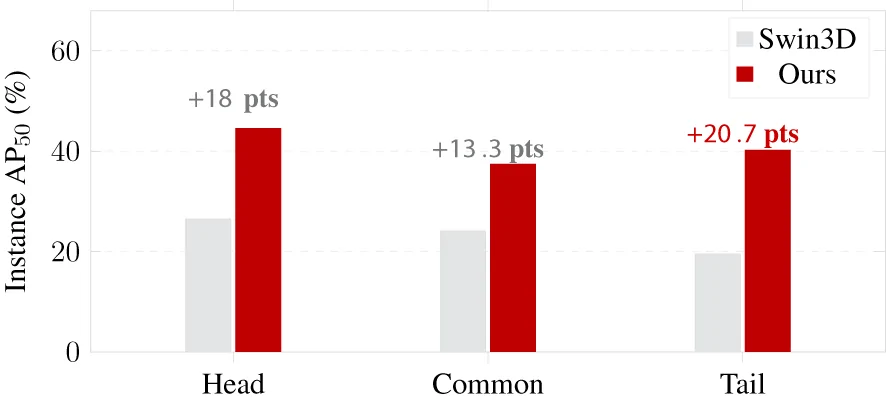
Instance AP_50_ across Head, Common, and Tail categories on ScanNet200. The figure compares the published Swin3D reference with the full model; the intermediate controlled configurations are reported in Table 11.

### 4.4 Memory and efficiency analysis

To isolate the contribution of the memory-efficient streaming integration, we compare the proposed block-wise implementation with a materialized cRSE implementation that stores the full pairwise bias tensor. Both implementations are evaluated on a single 24 GB RTX 4090 GPU with batch size 4 and the same window capacity.

As shown in Table 12, the materialized implementation at *N*_*w*_ = 32 serves as the ablation setting without streaming integration. Replacing it with the streaming implementation reduces peak online 3D-stage training memory from 18.3 to 13.6 GB, reduces the training step time from 564 to 461 ms, increases throughput from 1.80 to 2.20 iterations/s, and reduces inference latency from 142 to 127 ms per scene. These correspond to a 25.7% reduction in peak memory, an 18.3% reduction in training step time, and a 10.6% reduction in inference latency. The same streaming implementation supports window capacities of *N*_*w*_ = 48 and *N*_*w*_ = 64, with peak memory usage of 15.2 and 18.0 GB, respectively. Offline CLIP extraction, virtual-view rendering, 2D–3D alignment, and feature-cache storage are excluded from these measurements.

**Table 12:** Ablation of memory-efficient streaming integration and online 3D-stage efficiency measured on a single RTX 4090 GPU.

*N*_*w*_	Integration	Tile	Peak memory (GB)	Train step (ms)	Throughput (iter/s)	Inference (ms/scene)
32	Materialized	–	18.3	564	1.80	142
32	Streaming	16	**13.6**	**461**	**2.20**	**127**
48	Streaming	16	15.2	507	2.00	146
64	Streaming	16	18.0	677	1.50	181

The materialized row at *N*_*w*_ = 32 represents the implementation without streaming integration. All settings use batch size 4, and the streaming implementation uses a tile size of 16. Offline multimodal preprocessing is excluded.

Bold values indicate the best result within the corresponding dataset/task group or controlled comparison.

The online profile excludes multimodal preprocessing, whose costs are reported separately in Table 13. ScanNet and ScanNet200 require 19.3 and 19.9 s per scene, respectively, for loading the selected RGB-D frames, extracting CLIP features, and performing 2D–3D alignment. S3DIS requires 28.4 s because its virtual views must also be rendered. Cached features occupy 122–144 MB per scene, and 89.5%–94.8% of points receive at least one valid projection. The window statistics show irregular occupancy: as the window capacity increases from 32 to 64, the median token count increases from 19 to 35, while the 95th percentile reaches the capacity in all settings. The median unused capacity is computed as 1 − median(*N*)/*N*_*w*_ and is reported as an occupancy statistic, not as padding allocated by the streaming kernel.

**Table 13:** Offline multimodal preprocessing and sparse-window occupancy.

Dataset	Frame load/rendering (s)	CLIP (s)	Alignment (s)	Total (s)	Cache (MB/scene)	Valid points (%)	Valid views per covered point
(a) Offline preprocessing and feature coverage							
ScanNet	4.0	10.2	5.1	19.3	142	89.7	4.5
ScanNet200	4.2	10.4	5.3	19.9	144	89.5	4.5
S3DIS	14.5	9.3	4.6	28.4	122	94.8	5.1
**Window cap**	**P25 tokens**	**Median**	**P75**	**P95**	**Median unused capacity**		
(b) Sparse-window occupancy							
32	11	19	27	32	40.6%		
48	15	27	39	48	43.8%		
64	19	35	51	64	45.3%		

Panel (a) reports the measured per-scene summary; panel (b) summarizes tokens per window.

### 4.5 Qualitative results

Figure 4 illustrates representative ScanNet cases involving a small object, an ambiguous local boundary, and adjacent furniture with similar appearance. The middle column shows the Swin3D baseline; the red boxes identify the regions discussed in the text. Relative to that baseline, the full model better preserves the small region in these examples and reduces local label confusion.

**Figure 4:**
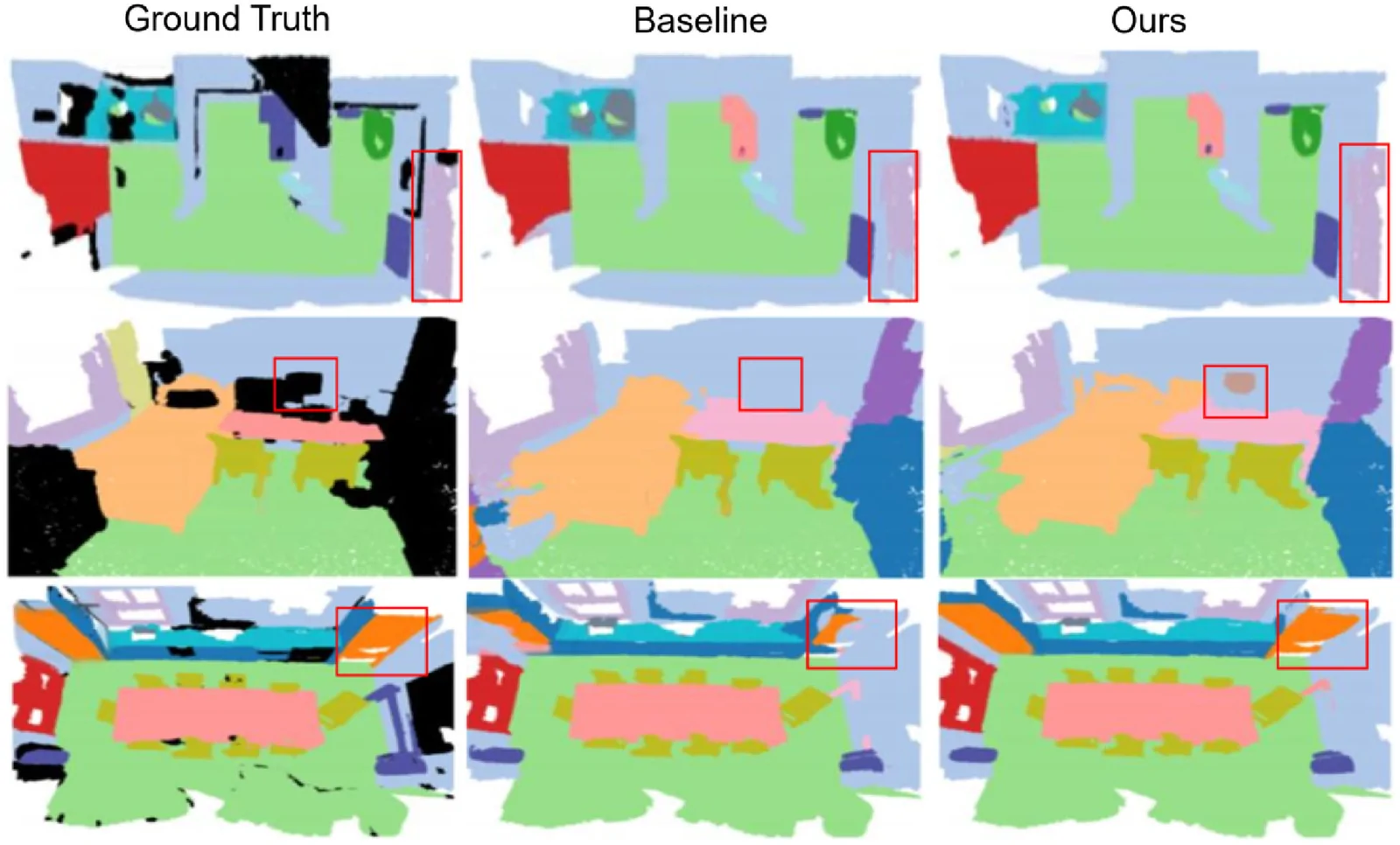
Qualitative ScanNet results. Columns show the ground truth, the Swin3D baseline, and the full model. Red boxes indicate representative regions discussed in the text.

To complement the ScanNet examples, Figure 5 presents qualitative semantic-segmentation results on S3DIS Area 5, including the input scenes, ground-truth annotations, and predictions from the full model.

**Figure 5:**
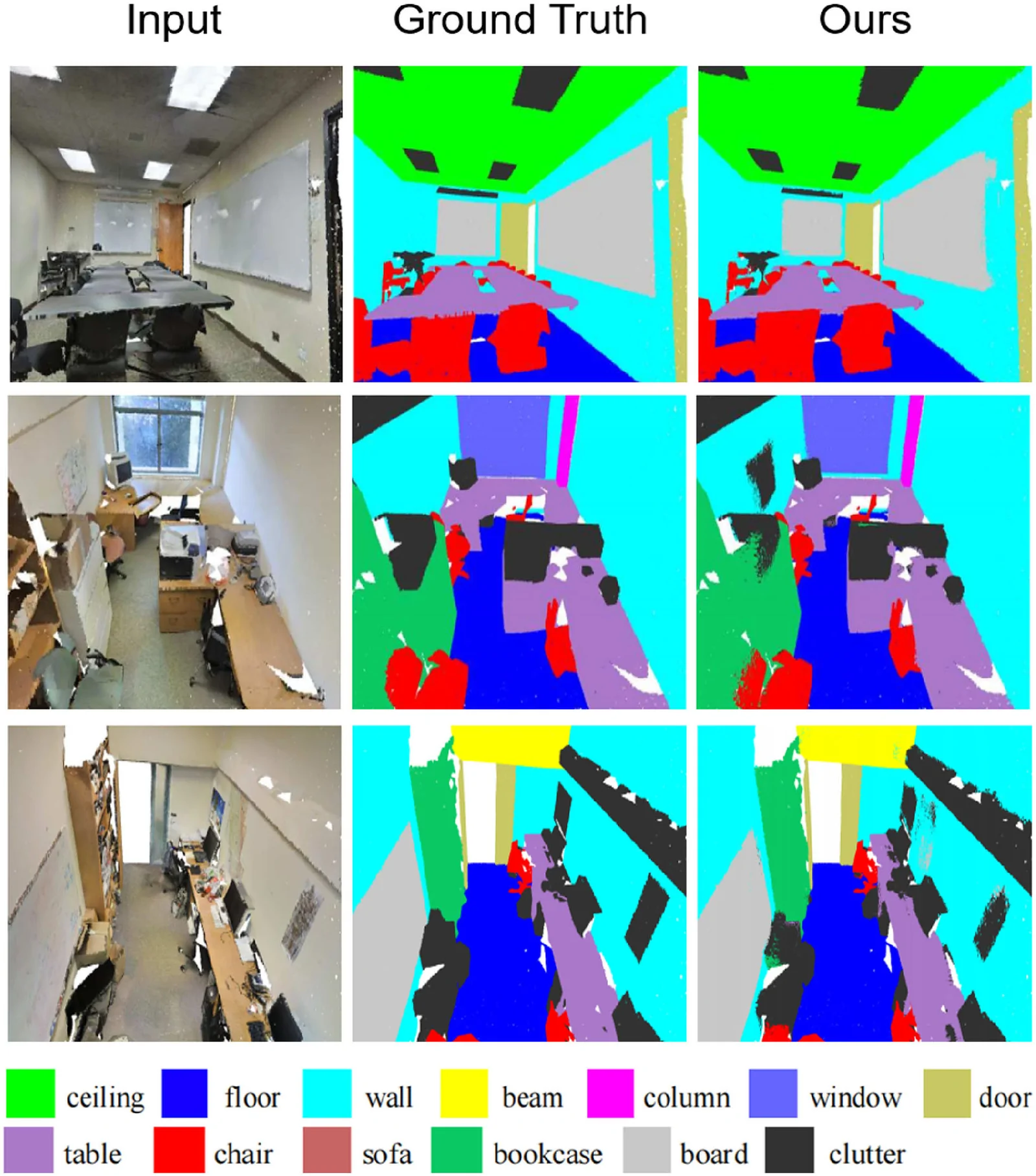
Qualitative semantic-segmentation results on S3DIS Area 5. Columns show the input scene, ground truth, and the full model.

To complement the semantic examples, Figure 6 shows representative ScanNet200 instance-segmentation results. The examples compare the input scene, ground-truth instance annotations, the baseline prediction, and the prediction of the full model. The highlighted regions illustrate cases involving small or visually ambiguous instances and show the differences between the two configurations.

**Figure 6:**
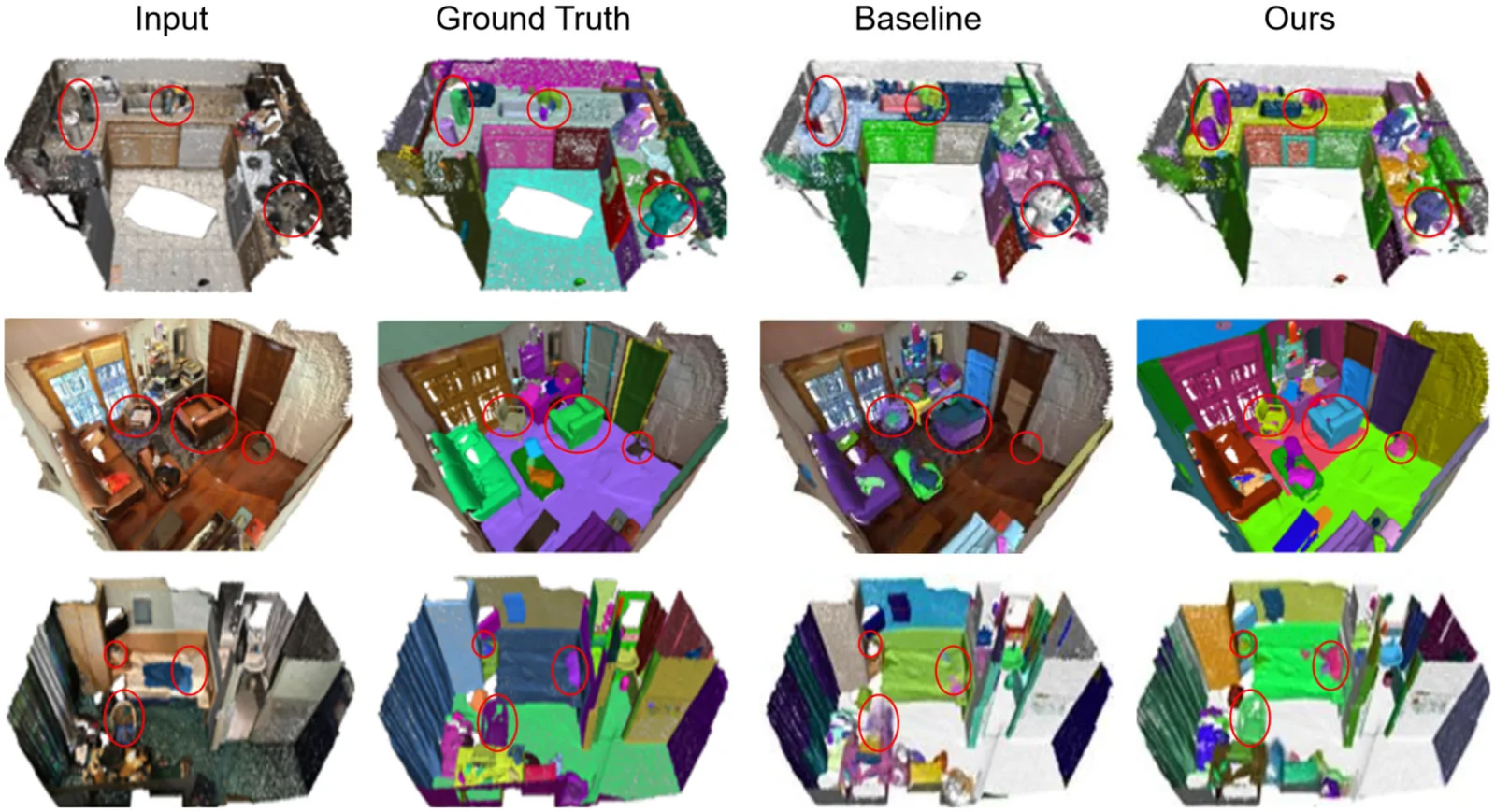
Representative qualitative instance-segmentation results on ScanNet200. The columns show the input scene, ground truth, the baseline prediction, and the full-model prediction. The highlighted regions indicate representative instance-level differences.

## 5 Conclusion

In this paper, we presented a CLIP-guided point-cloud segmentation framework for bridging the semantic-geometric gap in sparse 3D scene understanding. Dense CLIP features are aligned with 3D points through visibility-aware projection and view pooling to enrich sparse voxel tokens. Building on contextual relative position encoding, the proposed semantic-geometric extension adds a decoupled CLIP-induced discrepancy residual to geometry and appearance biases in local attention. Its block-wise implementation generates and consumes bias tiles during online softmax, avoiding full pairwise bias materialization. Experiments on ScanNet, ScanNet200, and S3DIS show that the largest performance gains arise from aligned CLIP input and structural semantic–geometric modeling, while the decoupled semantic residual provides a smaller complementary refinement. The full system also improves long-tail recognition and reduces the memory required for cRSE integration under the reported setting.

### 5.1 Limitations and future work

Although aligned CLIP semantics provide effective high-level cues for 3D point-cloud segmentation, the current framework still depends on the availability and quality of multi-view RGB observations, camera calibration, and 2D–3D alignment. Its performance may therefore be affected by inaccurate poses, occlusions, incomplete view coverage, or noisy depth consistency during visibility-aware projection. For ScanNet and ScanNet200, the aligned CLIP features are obtained from native posed RGB-D sequences. For S3DIS, however, posed RGB-D video sequences are not available in the same form, and the aligned CLIP features are obtained from virtual RGB-D views rendered from colored point clouds. Therefore, the S3DIS results should be interpreted under a rendered-view alignment setting rather than a native RGB-D sequence setting.

Another limitation is the system-level cost of multimodal preprocessing. The proposed streaming integration reduces the peak GPU memory of cRSE attention inside the 3D backbone, especially compared with naive pairwise bias materialization. CLIP feature extraction, virtual view rendering, feature storage, and 2D–3D alignment remain offline preprocessing steps; their measured time and cache footprint are reported in Table 13. Thus, the memory reduction refers specifically to semantic-geometric attention during 3D-network training rather than the total peak resource cost of an online end-to-end multimodal system.

In addition, the current empirical evaluation focuses on static indoor benchmarks. Future work will explore more robust cross-modal alignment strategies, efficient online or cached CLIP feature preprocessing pipelines, and extensions of the proposed memory-efficient semantic-geometric attention paradigm to large-scale outdoor environments such as autonomous driving point clouds. We will also investigate 3D-native foundation models to reduce the dependence on dense 2D-to-3D feature projection.

## Data Availability

The raw data supporting the conclusions of this article will be made available by the authors, without undue reservation.
