# Supplementary material for: Vision–language guided semantic-geometric transformer for memory-efficient 3D scene understanding
Source: Front Artif Intell. 2026 Sep 25;9:1945529. doi: 10.3389/frai.2026.1945529 (PMC13649730; doi:10.3389/frai.2026.1945529)
Supplement: Supplementary file 1 [file Data_Sheet_1.pdf]

# Supplementary Material

## 1 SCOPE AND REPRODUCIBILITY RECORD

This supplementary material gives the definitions and fixed settings used by the main manuscript. The reported online efficiency numbers refer to the 3D stage with cached CLIP features. Offline image encoding, virtual-view rendering, 2D–3D alignment, and cache construction are measured separately. The deterministic view-generation protocol, feature-cache procedure, training configuration, inference settings, and streaming attention implementation are described below.

**Table S1.** Configuration values explicitly specified by the manuscript.

| Item | Setting |
|---|---|
| Backbone | Four-stage hierarchical Swin3D-style encoder with alternating W-MSA3D and SW-MSA3D blocks |
| Encoder channels | $[C_1, C_2, C_3, C_4] = [48, 96, 192, 384]$ |
| Blocks per stage | $[D_1, D_2, D_3, D_4] = [2, 2, 6, 2]$ |
| Attention heads | $[H_1, H_2, H_3, H_4] = [3, 6, 12, 24]$ |
| Decoder | Four-stage sparse U-Net decoder with transposed sparse convolutions and skip connections |
| Voxel size | $v_s = 0.02\,\mathrm{m}$ |
| Window caps | $N_w \in \{32, 48, 64\}$; base setting $N_w = 32$ |
| Streaming tile | Key/value tile size 16 |
| Hardware | One RTX 4090 with 24 GB memory; batch size 4 |
| Optimizer | AdamW, learning rate $10^{-3}$, weight decay $5 \times 10^{-2}$, and cosine decay |
| Warm-up | 1500 iterations |
| Training duration | 300 epochs |
| Cache serialization | Scene-level `.npz` files indexed by scene identifier |
| Numerical-stability constant | $\epsilon = 10^{-6}$ |
| Loss weights | $\lambda_{\text{ins}} = 1.0$, $\lambda_{\text{obj}} = 1.0$, and $\lambda_{\text{emb}} = 1.0$ |
| Embedding margins | $\delta_v = 0.5$ and $\delta_d = 1.5$ |
| Random seeds | 42 by default; 41, 42, and 43 for the stability study |

Here, $N_s$, $C_s$, $D_s$, and $H_s$ denote the number of active sparse tokens, feature-channel width, number of blocks, and number of attention heads at encoder stage $s \in \{1, 2, 3, 4\}$, respectively. The values of $N_s$ are determined by the input scene and the voxelization or downsampling operation, and are not separately tuned hyperparameters. The decoder uses the corresponding fixed sparse U-Net configuration.

## 2 INSTANCE SEGMENTATION HEAD

The main paper keeps only the total objective because the instance head follows a standard offset–embedding–objectness design. For completeness, the decoded feature of voxel $\nu$ produces

$$\mathbf{o}_\nu = \mathrm{MLP}_{\text{off}}(\mathbf{d}_\nu), \quad \mathbf{h}_\nu = \mathrm{MLP}_{\text{emb}}(\mathbf{d}_\nu), \quad s_\nu^{\text{obj}} = \sigma\big(\mathrm{MLP}_{\text{obj}}(\mathbf{d}_\nu)\big)\,. \tag{S1}$$

Here, $\mathrm{MLP}_{\mathrm{off}}$, $\mathrm{MLP}_{\mathrm{emb}}$, and $\mathrm{MLP}_{\mathrm{obj}}$ are learned multilayer perceptron heads, and $\sigma(\cdot)$ is the sigmoid function. For a point $i$ assigned to voxel $\nu(i)$, the point-level predictions used in the losses and post-processing are obtained from the corresponding voxel predictions:

$$\mathbf{o}_i = \mathbf{o}_{\nu(i)}, \qquad \mathbf{h}_i = \mathbf{h}_{\nu(i)}, \qquad s_i^{\mathrm{obj}} = s_{\nu(i)}^{\mathrm{obj}}.$$

Let $\Omega_{\mathrm{fg}}$ denote the foreground points belonging to valid supervised instances, and let $\Omega_{\mathrm{sup}}$ denote all points with valid objectness labels. Ignored, unlabeled, and void points are excluded. Here, $\mathbf{o}_i^*$ denotes the ground-truth offset from point $i$ to the center of its assigned instance.

The offset loss is

$$\mathcal{L}_{\mathrm{off}} = \frac{1}{|\Omega_{\mathrm{fg}}|} \sum_{i \in \Omega_{\mathrm{fg}}} \|\mathbf{o}_i - \mathbf{o}_i^*\|_1. \tag{S2}$$

The objectness loss is

$$\mathcal{L}_{\mathrm{obj}} = -\frac{1}{|\Omega_{\mathrm{sup}}|} \sum_{i \in \Omega_{\mathrm{sup}}} \left[ y_i \log s_i^{\mathrm{obj}} + (1 - y_i) \log(1 - s_i^{\mathrm{obj}}) \right]. \tag{S3}$$

The discriminative embedding loss is

$$\mathcal{L}_{\mathrm{emb}} = \mathcal{L}_{\mathrm{var}} + \mathcal{L}_{\mathrm{dist}},$$

where

$$\mathcal{L}_{\mathrm{var}} = \frac{1}{M} \sum_{m=1}^{M} \frac{1}{|\mathcal{I}_m|} \sum_{i \in \mathcal{I}_m} \left[ \|\mathbf{h}_i - \bar{\mathbf{h}}_m\|_2 - \delta_v \right]_+^2, \tag{S4}$$

$$\mathcal{L}_{\mathrm{dist}} = \frac{1}{M(M-1)} \sum_{m_a=1}^{M} \sum_{\substack{m_b=1 \\ m_b \neq m_a}}^{M} \left[ 2\delta_d - \|\bar{\mathbf{h}}_{m_a} - \bar{\mathbf{h}}_{m_b}\|_2 \right]_+^2. \tag{S5}$$

Here, $y_i \in \{0, 1\}$ is the binary objectness target, $M$ is the number of supervised instances, $\mathcal{I}_m$ denotes the set of points belonging to supervised instance $m$, $\bar{\mathbf{h}}_m$ is the mean embedding of instance $\mathcal{I}_m$, and $[x]_+ = \max(x, 0)$. We set the intra-instance variance margin to $\delta_v = 0.5$ and the inter-instance separation margin to $\delta_d = 1.5$ in all experiments. When fewer than two supervised instances are present, we set $\mathcal{L}_{\mathrm{dist}} = 0$.

## 3 INSTANCE INFERENCE AND POST-PROCESSING

Voxel predictions are mapped to raw points through the deterministic point-to-voxel correspondence. Points with objectness below 0.3 are discarded. Remaining points are shifted by $\hat{\mathbf{x}}_i = \mathbf{x}_i + \mathbf{o}_i$ and grouped in the normalized joint space

$$\mathbf{q}_i = [\hat{\mathbf{x}}_i / \tau_x \,\|\, \mathbf{h}_i / \tau_h], \tag{S6}$$

where $\tau_x = 0.03\,\mathrm{m}$, $\tau_h = 1.0$, $\|$ denotes channel-wise concatenation and the grouping radius is 1.0.

## 4 CLIP EXTRACTION AND 2D–3D ALIGNMENT

For ScanNet and ScanNet200, frames with invalid camera poses or missing depth maps are removed, and the remaining frames are ordered by temporal index. Given $M$ valid frames, we select 32 approximately uniformly spaced frames using

$$i_k = \mathrm{round}\left(\frac{k(M-1)}{31}\right), \qquad k = 0, \ldots, 31. \tag{S7}$$

The resulting frame lists are fixed and shared by all experiments.

For S3DIS, each room is first normalized according to its colored point-cloud bounding box. Exactly 32 deterministic candidate camera poses are generated for each room. The camera positions are defined in normalized room coordinates by

$$\hat{\mathbf{p}}_{a,\ell} = \Big(0.5 + 0.45\cos\theta_a,\ 0.5 + 0.45\sin\theta_a,\ \hat{h}_\ell\Big), \tag{S8}$$

where

$$\theta_a = \frac{2\pi a}{8}, \qquad a = 0, \ldots, 7, \tag{S9}$$

and

$$\hat{h}_\ell \in \{0.25,\ 0.45,\ 0.65,\ 0.85\}, \qquad \ell = 0, \ldots, 3. \tag{S10}$$

Each camera is oriented toward the room center. The same normalized camera layout and fixed camera intrinsics are used for all rooms.

Let $N_{\mathrm{room}}$ denote the number of points in a room. The projected-point coverage of candidate view $v$ is defined as

$$r_{\mathrm{cov}}^{(v)} = \frac{\left|\left\{i \in \{1, \ldots, N_{\mathrm{room}}\} : \mathbf{u}_i^{(v)} \in \Omega_{\mathrm{img}},\ m_i^{(v)} = 1\right\}\right|}{N_{\mathrm{room}}}, \tag{S11}$$

where $\mathbf{u}_i^{(v)}$ is the projected image coordinate defined in Eq. (1) of the main manuscript, $\Omega_{\mathrm{img}}$ is the image domain, and $m_i^{(v)} = 1$ indicates that the projection passes the depth-consistency test. Candidate views with

$$r_{\mathrm{cov}}^{(v)} < \tau_{\mathrm{cov}}, \qquad \tau_{\mathrm{cov}} = 0.05, \tag{S12}$$

are excluded from feature alignment. Thus, 32 candidate views are generated for each room, while the number of valid views used for a particular room may be smaller after coverage filtering.

CLIP ViT-L/14 processes each $336 \times 336$ image into a $24 \times 24$ grid of 1024-dimensional patch features, excluding the global token. Features are $\ell_2$-normalized and bilinearly sampled at projected point locations. A projection is valid only when it lies inside the image and its depth discrepancy is below $0.05\,\mathrm{m}$. Features from valid views are averaged using uniform weights. Points without valid observations receive a zero placeholder and a separate validity mask. Point-aligned CLIP features are stored in half precision. Each scene-level cache preserves the original point ordering and stores the point-aligned CLIP feature tensor $\mathbf{F}_{\mathrm{clip}} \in \mathbb{R}^{N\times 1024}$ in half precision together with the binary point-level validity vector $\mathbf{m}_{\mathrm{clip}} \in \{0,1\}^N$, whose $i$-th entry satisfies

$$[\mathbf{m}_{\mathrm{clip}}]_i = \rho_i^{\mathrm{clip}}.$$

**Table S2.** Deterministic virtual-view configuration for S3DIS.

| Parameter | Setting |
|---|---|
| Candidate views | 32 deterministic views per room |
| Camera coordinate system | Normalized room bounding box |
| Azimuth angles | $\theta_a = 2\pi a/8,\ a = 0, \ldots, 7$ |
| Camera heights | $\{0.25,\ 0.45,\ 0.65,\ 0.85\}$ in normalized room coordinates |
| Camera positions | $\hat{\mathbf{p}}_{a,\ell} = (0.5 + 0.45\cos\theta_a,\ 0.5 + 0.45\sin\theta_a,\ \hat{h}_\ell)$ |
| Camera orientation | Toward the room center |
| Image resolution | $336 \times 336$ |
| Focal lengths | $f_x = f_y = 168$ |
| Principal point | $(c_x, c_y) = (167.5, 167.5)$ |
| Depth rendering | Z-buffer rendering |
| Coverage threshold | $\tau_{\text{cov}} = 0.05$ |
| Depth-consistency threshold | $0.05\,\text{m}$ |

Thus, $[\mathbf{m}_{\text{clip}}]_i = 1$ if point $i$ receives at least one valid CLIP observation from the selected views. The cache is serialized as a scene-level `.npz` file indexed by the scene identifier.

## 5 END-TO-END EXPERIMENTAL PROTOCOL

The complete pipeline is executed in the following fixed order:

1. Load posed RGB-D frames for ScanNet and ScanNet200. Remove frames with invalid camera poses or missing depth maps, order the remaining frames by temporal index, and select 32 approximately uniformly spaced frames using Eq. (S7).
2. For S3DIS, normalize each room according to its colored point-cloud bounding box and generate the 32 deterministic candidate views specified in Section 4.
3. Render the S3DIS candidate views using fixed camera intrinsics and z-buffer visibility. Candidate views with projected-point coverage below $\tau_{\text{cov}} = 0.05$ are excluded from feature alignment.
4. Resize every RGB image to $336 \times 336$ and apply the frozen CLIP ViT-L/14 image encoder. The last-layer patch tokens are reshaped into a normalized $24 \times 24 \times 1024$ feature map.
5. Project each 3D point using the corresponding camera intrinsics and extrinsics. A projection is retained only when it lies inside the image and its depth discrepancy is below $0.05\,\text{m}$.
6. Bilinearly sample the CLIP features and uniformly average valid features across views. Points without valid projections receive a zero feature together with a binary validity flag.
7. Cache the point-aligned CLIP features and validity masks in half precision. The same fixed caches are used during 3D training and inference, and the CLIP encoder is not executed online.
8. Voxelize the point cloud using $v_s = 0.02\,\text{m}$ and process the sparse tokens with the Swin3D-style backbone. The base window capacity is $N_w = 32$, and the streaming implementation uses key/value tiles of size 16.
9. Train for 300 epochs using AdamW with learning rate $10^{-3}$, weight decay $5 \times 10^{-2}$, cosine decay, 1500 warm-up iterations, and batch size 4. Seed 42 is used by default, while seeds 41, 42, and 43 are used for the stability experiments.

10. Apply the fixed semantic and instance inference procedures. All controlled variants use identical frame lists, camera layouts, caches, augmentations, training schedules, instance heads, and post-processing parameters.

Online efficiency measurements cover only the 3D stage with cached CLIP features. Virtual-view rendering, CLIP extraction, 2D–3D alignment, and cache construction are measured separately.

The implementation and configuration files, including the deterministic camera layout and preprocessing settings, will be released in an archived repository upon acceptance.

## 6 STREAMING ATTENTION PROCEDURE

For a fixed query vector $\mathbf{q}$, the keys and values are partitioned into $T$ tiles. Tile $t$ contains the key matrix $\mathbf{K}_t$, value matrix $\mathbf{V}_t$, and cRSE-bias vector $\mathbf{B}_t^{\mathrm{cRSE}}$. Its logit vector is

$$\boldsymbol{\alpha}_t = \mathbf{q}\mathbf{K}_t^{\top}/\sqrt{d} + \mathbf{B}_t^{\mathrm{cRSE}},$$

where $d$ is the per-head feature dimension. The index $j$ runs over the entries in tile $t$, while $m_t$, $\ell_t$, and $\mathbf{a}_t$ denote the running maximum, normalization factor, and value accumulator after processing tile $t$, respectively.

$$\begin{aligned} m_t &= \max(m_{t-1}, \max \boldsymbol{\alpha}_t), \\ \ell_t &= e^{m_{t-1}-m_t}\ell_{t-1} + \sum_j e^{\alpha_{t,j}-m_t}, \\ \mathbf{a}_t &= e^{m_{t-1}-m_t}\mathbf{a}_{t-1} + \sum_j e^{\alpha_{t,j}-m_t}\mathbf{v}_{t,j}. \end{aligned} \tag{S13}$$

Here, $\alpha_{t,j}$ and $\mathbf{v}_{t,j}$ are the $j$-th logit and value vector in tile $t$, and $T$ is the total number of tiles. The initialization is $m_0 = -\infty$, $\ell_0 = 0$, and $\mathbf{a}_0 = \mathbf{0}$; the output is $\mathbf{o} = \mathbf{a}_T/\ell_T$. The cRSE tile is discarded after the update.
